# Reclassification of the Etiology of Infant Mortality With Whole-Genome Sequencing

**DOI:** 10.1001/jamanetworkopen.2022.54069

**Published:** 2023-02-09

**Authors:** Mallory J. Owen, Meredith S. Wright, Sergey Batalov, Yonghyun Kwon, Yan Ding, Kevin K. Chau, Shimul Chowdhury, Nathaly M. Sweeney, Elizabeth Kiernan, Andrew Richardson, Emily Batton, Rebecca J. Baer, Gretchen Bandoli, Joseph G. Gleeson, Matthew Bainbridge, Christina D. Chambers, Stephen F. Kingsmore

**Affiliations:** 1 Rady Children’s Institute for Genomic Medicine, Rady Children’s Hospital, San Diego, California; 2 Department of Pediatrics, University of California, San Diego, La Jolla; 3 California Preterm Birth Initiative, University of California, San Francisco

## Abstract

**Importance:**

Understanding the causes of infant mortality shapes public health, surveillance, and research investments. However, the association of single-locus (mendelian) genetic diseases with infant mortality is poorly understood.

**Objective:**

To determine the association of genetic diseases with infant mortality.

**Design, Setting, and Participants:**

This cohort study was conducted at a large pediatric hospital system in San Diego County (California) and included 546 infants (112 infant deaths [20.5%] and 434 infants [79.5%] with acute illness who survived; age, 0 to 1 year) who underwent diagnostic whole-genome sequencing (WGS) between January 2015 and December 2020. Data analysis was conducted between 2015 and 2022.

**Exposure:**

Infants underwent WGS either premortem or postmortem with semiautomated phenotyping and diagnostic interpretation.

**Main Outcomes and Measures:**

Proportion of infant deaths associated with single-locus genetic diseases.

**Results:**

Among 112 infant deaths (54 girls [48.2%]; 8 [7.1%] African American or Black, 1 [0.9%] American Indian or Alaska Native, 8 [7.1%] Asian, 48 [42.9%] Hispanic, 1 [0.9%] Native Hawaiian or Pacific Islander, and 34 [30.4%] White infants) in San Diego County between 2015 and 2020, single-locus genetic diseases were the most common identifiable cause of infant mortality, with 47 genetic diseases identified in 46 infants (41%). Thirty-nine (83%) of these diseases had been previously reported to be associated with childhood mortality. Twenty-eight death certificates (62%) for 45 of the 46 infants did not mention a genetic etiology. Treatments that can improve outcomes were available for 14 (30%) of the genetic diseases. In 5 of 7 infants in whom genetic diseases were identified postmortem, death might have been avoided had rapid, diagnostic WGS been performed at time of symptom onset or regional intensive care unit admission.

**Conclusions and Relevance:**

In this cohort study of 112 infant deaths, the association of genetic diseases with infant mortality was higher than previously recognized. Strategies to increase neonatal diagnosis of genetic diseases and immediately implement treatment may decrease infant mortality. Additional study is required to explore the generalizability of these findings and measure reduction in infant mortality.

## Key Points

**Question:**

What proportion of infant mortality is explained by genetic diseases?

**Findings:**

In this cohort study of 112 infant deaths, single-locus genetic diseases were the most common antecedent of infant mortality (41%). Treatments positively associated with outcomes were available for 30% of these genetic diseases.

**Meaning:**

The study results suggest that because treatable genetic diseases are associated with considerable infant mortality, strategies for neonatal diagnosis may be associated with decreased infant mortality.

## Teaser

This cohort study examines the association of genetic diseases with infant mortality at a large pediatric hospital system in San Diego County, California.

## Introduction

Infant mortality in the US remains high (approximately 1 in 200 live births).^1,2^ Congenital malformations/chromosomal abnormalities (malformations: *International Classification of Diseases, Tenth Revision [ICD-10]* codes Q00-Q99) have been the leading cause of US infant death for more than 50 years.^1,2,3,4,5^ National vital statistics indicate that malformations account for more than one-fifth of infant deaths, followed by preterm birth/low birth weight (prematurity), pregnancy complications, and sudden infant death syndrome (SIDS).^1,2^ Accurate etiologic classification of infant mortality is important for families and the public. Aggregate county, state, and national statistics inform prioritization of public health and research programs.^6,7^ For example, the Back to Sleep/Safe to Sleep public health programs have been associated with decreased mortality that is associated with SIDS.^8,9,10^ Improvements in obstetric and neonatal care have been associated with decreased mortality that is associated with prematurity.^1,2,3,4,5^

Prior etiologic studies of infant mortality are generally retrospective and based on electronic health record (EHR) and death certificate review,^6,7^ potentially leading to underdiagnosis of genetic diseases. Furthermore, at least 30% of death certificates have inaccuracies.^11,12,13,14^ The effect of such imprecision could be large since many genetic diseases have treatments that can improve outcomes, and undiagnosed genetic diseases often recur within families, causing preventable deaths.^15,16,17,18,19,20,21,22,23,24,25,26,27,28,29,30,31,32^ Early implementation of genomic sequencing could improve understanding about causes and suggest novel strategies to reduce infant mortality. Genomic sequencing has shown that single-locus genetic diseases are a leading cause of some categories of infant deaths, such as SIDS, but their association with overall infant mortality has not been well quantified.^15,16,17,18,19,20,21,22,23,24,25,26,27,28^ Rady Children’s health system adopted rapid, diagnostic whole-genome sequencing (WGS) in the care of infants in intensive care with diseases of unknown etiology in 2015.^26,27,28,29,30,31,32,33^ In this article, we report results of WGS in a cohort of infant deaths that occurred in a single pediatric hospital system from 2015 to 2020.

## Methods

### Study Design

This was a retrospective cohort study with 3 observational arms (Figure). It was approved by the institutional review boards of the University of California, San Diego and San Francisco. They did not consider postmortem WGS to be human participants research and provided a waiver of informed consent for the study. All participants underwent standard genetic testing as clinically indicated. Premortem WGS was performed with informed parental consent either as a clinical diagnostic test or in research protocols (ClinicalTrials.gov: NCT03211039, NCT02917460, and NCT03385876).^29,30,31,32,33^ The indications for infant WGS were those published by Blue Shield-California. Postmortem WGS was performed for all infants with blood sample retains that were archived in the Rady Children’s biorepository. Administrative data were obtained from public sources in the San Diego Study of Mothers and Infants from 2015 to 2019. Data from 2020 were not yet available. All data have been deidentified. Results were reported according to the Strengthening the Reporting of Observational Studies in Epidemiology (STROBE) reporting guidelines for reporting observational studies.

**Figure.**
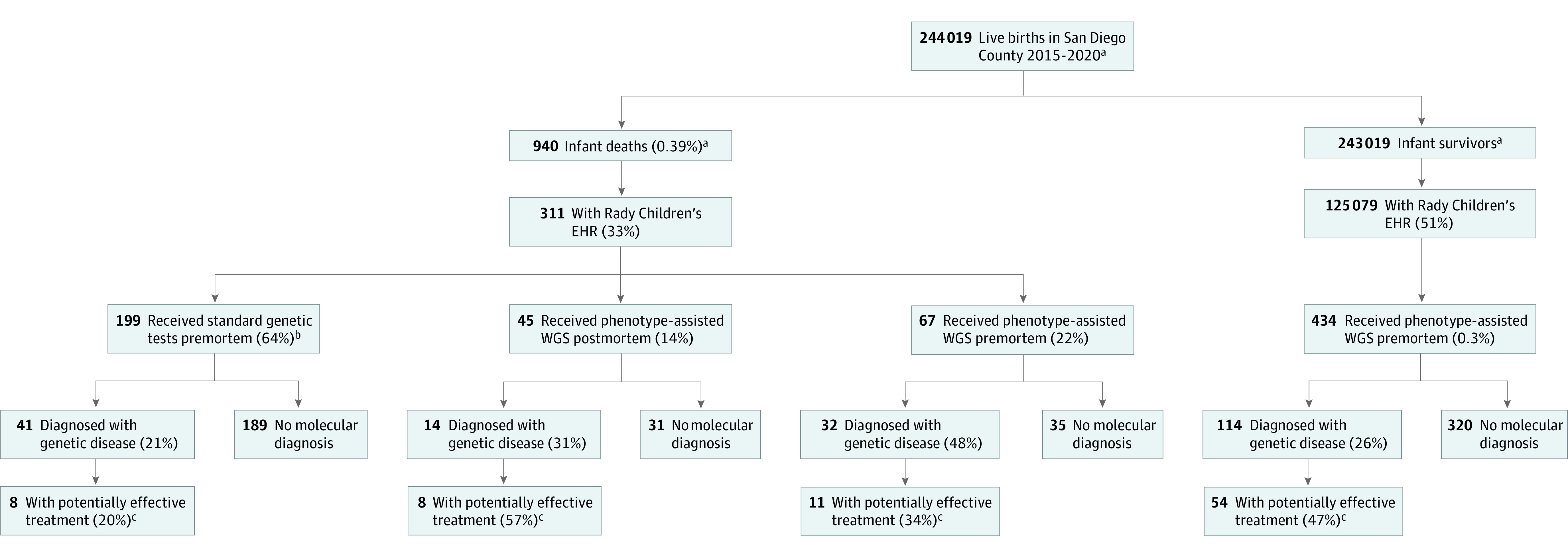
Flow Diagram of the Observational Study of Infant Survivors and Infants Who Died Who Underwent Whole-Genome Sequencing (WGS) for Diagnosis of Genetic Diseases During Care at Rady Children’s Hospital, San Diego, California, from 2015 to 2020 The left side of the diagram represents 45 infant deaths who received WGS postmortem and 67 who received rapid WGS for diagnosis of a suspected genetic disease during intensive care unit (ICU) admission. The right side of the diagram represents the control group, comprising infant survivors who received rapid WGS for diagnosis of a suspected genetic disease during ICU admission. ^a^2020 deaths projected from average of 2015 to 2019 total deaths. ^b^Standard genetic tests included chromosomal microarray, gene, and panel sequencing. ^c^The efficacy of interventions for diseases associated with infant mortality in this study was adjudicated with the Genome-to-Treatment system and a similar online compendium of treatable genetic disorders.^34,35^

### Race and Ethnicity

Infant race and ethnicity were classified by parents and extracted from the EHR. Race and ethnicity options were defined by the EHR. Race and ethnicity were assessed since to our knowledge there is a paucity of information regarding the diagnostic use of genome sequencing in racial and ethnic minority groups and a dearth of reference genome sequences from racial and ethnic minority groups, from which the racial-specific and ethnicity- specific allele frequencies used in genome interpretation are determined. Infants with a racial and ethnic classification of “other” were those whose parents did not categorize them as American Indian or Alaska Native, Asian, Black or African American, Hispanic, Native Hawaiian or Pacific Islander, non-Hispanic, or White and included multiracial infants.

### WGS and Genetic Disease Identification

Whole-genome sequencing was performed from blood samples or dried blood spots, as described.^26,27,28,29,30,31,32,33,36^ Premortem WGS was performed with parent-child duos or trios for whom parental samples were available, and parents gave permission for their genomes to be sequenced. Postmortem WGS was of singleton proband samples. Whole-genome sequencing was performed with 2 × 101 nt and a depth of more than 30 fold (Illumina). Read alignment to *GRCh37* and variant diplotype identification was with DRAGEN (Illumina) and included copy number and structural variant identification. Semiautomated interpretation was performed using MOON (InVitae), GEM, and Enterprise (Fabric Genomics) as described.^37,38^ Inputs were variant call files, manually curated lists of observed human phenotype ontology terms, and metadata. Reportable diplotypes were identified by filtering and ranking disease phenotype match, variant pathogenicity, and rarity using decision trees, bayesian models, neural networks, and natural language processing and classified according to American College of Medical Genetics and Genomics guidelines by molecular laboratory directors.^37^ Variants of uncertain significance were only included if located in a gene that was casually associated with a genetic disease whose expected clinical features in infancy clearly overlapped the observed phenotypes in the proband and was known to be associated with infant mortality (variants of uncertain significance suspicious). Whole-genome sequencing was interpreted once. Reanalysis of negative cases was not performed.

### Adjudication of Efficacy of Interventions for Genetic Diseases

An expert panel undertook a structured adjudication of the indications, contraindications, efficacy, and evidence of efficacy of 9911 drug, device, dietary, and surgical interventions for 563 severe childhood genetic diseases, as described previously.^37^ Of these, 421 diseases and 1527 effective interventions (15%) were retained and integrated with 13 genetic disease information resources (Genome-to Treatment: https://gtrx.radygenomiclab.com). For diseases associated with infant mortality, the efficacy of interventions was adjudicated with the Genome-to-Treatment system and a similar online compendium of treatable genetic disorders.^38,39^

### Statistical Analyses

Groups were compared with χ^2^ and Fisher exact tests. Unadjusted *P* values less than .01 were considered significant.

## Results

### Infant Cohorts and Demographic Characteristics

Between 2015 and 2020, 940 infants (0.39% of live births) died in San Diego County, California, of which 311 (33%) had Rady Children’s Hospital or University of California, San Diego health records (Figure). Of these, 112 (36%) underwent WGS either as a rapid, inpatient diagnostic test (67 [59.8%]) or postmortem using archived dried blood spots (45 [50.2%]).^29,30,31,32,33^ Whole-genome sequencing identified 47 single-locus genetic diseases in 46 infant deaths (41%) (Table 1). Genetic diseases were identified by premortem, rapid WGS in 26 infants (55%), other premortem genetic tests in 6 (13%) (with WGS confirmation), and postmortem WGS in 14 (30%). Thirty-nine of the 47 (83%) genetic diseases had previously been reported to be associated with childhood mortality. A literature review identified treatments that can improve outcomes for 19of the 47 (51%) genetic diseases (Table 1).^38,39,40,41^

**Table 1.** Forty-seven Genetic Diseases Identified by WGS in 46 of 112 Infant Deaths in San Diego

Patient No./Sex	Affected locus	Variant 1	Variant 2	Inheritance	Condition	Zygosity	ACMG classification	Clinical course	Dx premortem or postmortem	Disease causes of infant death	Effective Rx potentially available
101/F	*KMT2D*	c.5546G>A, p.G1849E	NA	AD	Kabuki syn 1	Het	VUS	N	Post	Yes	No
107/M	*PKHD1*	c.10219C>T, p.Q3407*	c.107C>T, p.T36M	AR	Polycystic kidney dis 4	CH	P/P	N	Pre	Yes	Yes
108/F	1q31.1q42.2 Dup	Chr1:192411878-235102223 dup	NA	AD	Partial trisomy chr1q	Tri	P	N	Pre	Yes	No
114/M	Trisomy 21	Chr21:1-48 129 895 dup	NA	AD	Trisomy 21	Tri	P	N	Post	Yes	No
119/M	*SCN1A*	c.1625G>A, p.R542Q	c.2057A>C, p.E686A	AR	*DEE6B*/ Dravet syn	Het	VUS	N	Post	Yes	Yes
121/M	17q11.2 (NF1) del	Chr17:29 001 242-30 368 486 del	NA	AD	Neurofibromatosis 1	Het	P	KC	Pre	Yes	Yes
122/F	*NFKB1*	c.1576G>A, p.V526M	NA	AD	Common variable immunodef 12	Het	VUS	N	Post	Yes	Yes
124/F	*SUOX*	c.1390_1391delCT, p.L464fs	NA	AR	Sulfite oxidase def	Hom	P	N	Post	Yes	No
126/M/	*NIPBL*	c.5455C>T, p.R1819*	NA	AD	Cornelia De Lange syn 1	Het	P	KC	Pre	Yes	No
128/F	*CHD7*	c.1058delT, p.F353Sfs	NA	AD	CHARGE syn	Het	P	KC	Pre	Yes	Yes
133/M	*AUTS2*	c.1180dupT, p.Y394fs	NA	AD	Intellectual dv dis 26	Het	P	KC	Post	No	No
138/F	*RYR1*	c.11763C>A, p.Y3921*	NA	AD	Muscle central core dis	Het	VUS	N	Post	Yes	No
141/F	*GATA6*	c.1480A>T, p.K494*	NA	AD	Pancreatic agenesis and congenital heart defects	Het	P	N	Post	Yes	Yes
142/F	*FGFR3*	c.742C>T, p.R248C	NA	AD	Thanatophoric dwarfism	Het	P	KC	Pre	Yes	No
146/M	*EPHB4*	c.1170G>A, p.W390*	NA	AD	Lymphatic malformation 7	Het	VUS	FP	Post	Yes	No
153/M	14q31.2q32.2 del	Chr14:84783523-96907490 del	NA	AD	Chr 14q31.2q32.2 del syn	Het	P	N	Pre	Yes	No
155/F	*CHD7*	c.496C>T, p.Q166*	NA	AD	CHARGE syn	Het	P	N	Pre	Yes	Yes
158/F	17q12 del	Chr17:34759401-36284600 del	NA	AD	Chr 17q12 del syn	Het	P	N	Pre	Yes	No
160/M	*TAZ*	c.811C>T, p.Q271*	NA	XLR	Barth syn	Hem	P	KC	Pre	Yes	Yes
162/F	*SOX9*	c.196G>T, p.E66*	NA	AD	Campomelic dysplasia with sex reversal	Het	P	KC	Pre	Yes	No
166/F	*ARID1B*	c.3096_3100CAAAG,p.K1033Rfs	NA	AD	Coffin-Siris syn 1	Het	P	KC	Pre	Yes	No
168/M	12q21.33q22 del	Chr12:91 455 327-95 023 446	NA	AD	12q21.33q22 del syn	Het	VUS	KC	Pre	Yes	No
170/F	*ANK2*	c.1574C>T, p.A525V	NA	AD	Long QT syn 4	Het	VUS	FP	Post	Yes	Yes
171/M=	*FBN1*	c.3211A>G, p.I1071V		AD	Marfan syn	Het	LP	KC	Pre	Yes	No
173/M=	*B3GALT6*	c.950C>T, p.P317L	c.122C>T, p.A41V	AR	Spondylodysplastic Ehlers-Danlos syn 2	Het	VUS x2	KC	Post	Yes	No
177/F/	*SDHA*	c.1795-1G>T	c.480T>G p.F160L	AR	Mitochondrial complex II def	Het	P/LP	KC	Pre	Yes	No
178/M	*TRNT1* (1); *GPD1L* (2)	(1) c.443C>T, p.A148V	(2) c.839C>T, p.A280V	AR/AD	SIFD, Brugada syn 2	Hom/ het	P/LP	KC	Pre	Yes	Yes Yes
181/M	*NDUFV1*	c.383G>A, p.R128Q	c.166T>C, p.S56P	AR	Mitochondrial complex I def	Het	LP/LP	N	Post	Yes	No
182/F	*MBD5*	c.2426C>T, p.P809L	NA	AD	AD mental retardation 1	Het	VUS	N	Post	No	No
183/F	*CACNA1C*	c.1864G>A, p.V622I	NA	AD	Long QT syn 8	Het	VUS	FP	Post	Yes	Yes
184/F	*TAB1* ^a^	c.1084G>A, p.V362M	NA	AR	Novel	Hom	VUS	N	Post	No	No
188/M	*SAMD9*	c.2611G>T, p.E871*	NA	AD	MIRAGE Syn.	Het	LP	N	Post	Yes	Yes
189/M	*COL6A3*	c.3986G>A, p.G1329E	NA	AD	Ullrich congenital muscular dystrophy 1	Het	VUS	N	Post	No	No
191/F	*TAB1*	c.1084G>A, p.V362M	NA	AR	Novel	Hom	VUS	FP	Post	No	No
192/F	*PCDH19*	c.2559C>G, p.F853L	NA	AD	DEE9	Het	VUS	N	Post	No	Yes
194/F	*COQ2*	c.590G>A, p.R197H	c.151A>G, p.M51V	AR	Primary coenzyme Q10 def	Het	LP/VUS	KC	Pre	Yes	Yes
198/F	Trisomy 22	Chr22: 51989170-67133222 dup	NA	AD	Chr 22 mosaic partial trisomy	Tri	P	N	Pre	Yes	No
200/M	TTN	c.38767A>T, p.K12923*	c.96377G>A, p.W32126*	AR	Salih myopathy	Het	LP/LP	KC	Pre	Yes	No
201/M	*MOCS1*	c.377G>A, p.G126D	c.*7 + 5G>A	AR	Molybdenum cofactor def	Het	LP/LP	N	Pre	Yes	Yes
203/F	Chr22q11.21 Del	Chr22:18851101-21481300 del	NA	AD	DiGeorge syn	Het	P	KC	Pre	Yes	No
205/M	*IKBKG*	c.665_666delAG, p.E222Gfs	NA	XLR	XL ectodermal dysplasia and immunodef 1	Hem	LP	N	Pre	Yes	Yes
207/F	11p15.5-q25	Chr15:1-135086622	NA	AD	Beckwith-Wiedemann syn	Het	P	KC	Pre	Yes	Yes
	Mosaic del										
209/F	*UBE3A*	c.365T>G, p.F122C	NA	AD	Angelman syn	Het	VUS	N	Pre	No	No
210/M	*SIX3*	c.801_806 + 28del	NA	AD	Holoprosencephaly 2	Het	P	N	Post	Yes	No
211/F	18p11.32q23 Dup	Chr18:1-78077248 dup	NA	AD	Edwards syn	Tri	P	N	Pre	Yes	No
212/M	*PPA2*	c.514G>A, p.E172K	c.442A>T, p.T148S	AR	Infantile sudden cardiac failure	Het	P/LP	KC	Post	Yes	Yes

Abbreviations: AD, autosomal dominant; AR, autosomal recessive; chr, chromosome; CHARGE, coloboma, heart defect, choanal atresia, retarded growth and development, genital hypoplasia, ear anomalies; DEE, developmental epileptic encephalopathy; def, deficiency; del, deletion; dis, disease; dv, development; Dx, diagnosis; FP, first presentation; hem, hemizygous; het, heterozygous; hom, homozygous; KC, known condition; LP, likely pathogenic; MIRAGE, myelodysplasia, infection, restriction of growth, adrenal hypoplasia, genital phenotypes, and enteropathy; N, neonatal; NA, not applicable; P, pathogenic; Rx, prescription; SIFD, sideroblastic anemia with B-cell immunodeficiency, periodic fevers, and developmental delay; syn, syndrome; VUS, variant of uncertain significance.

^a^ The candidacy of *TAB1* as a cause of infant death will be presented elsewhere.

Of 21 maternal and infant characteristics examined, 5 known risk factors for infant death differed significantly between the 46 infant deaths associated with genetic diseases and 66 without (Table 2). Premature birth, placental abruption, and maternal infection were more common in infant deaths without genetic diseases, and polyhydramnios was more common in genetic disease–associated deaths.

**Table 2.** Demographic and Clinical Characteristics of 112 Infants in San Diego County Who Died Who Received WGS^a^

Characteristic	Infant, No. (%)			*P* value
	Deaths	Deaths without genetic disease	Deaths with genetic disease	
Participants	112	66 (58.9)	46 (41.1)	NA
Sex				
Female	54 (48.2)	30 (45.5)	24 (52.2)	.48
Male	58 (51.8)	36 (54.5)	22 (47.8)	
Race and ethnicity^b^				
African American or Black	8 (7.1)	4 (6.1)	4 (8.7)	.33
American Indian or Alaska Native	1 (0.9)	0	1 (2.2)	
Asian	8 (7.1)	7 (10.6)	1 (2.2)	
Hispanic	48 (42.9)	30 (45.5)	18 (39.1)	
Native Hawaiian or Pacific Islander	1 (0.9)	1 (1.5)	0	
White non-Hispanic	34 (30.4)	19 (28.8)	15 (32.6)	
Other	12 (10.7)	5 (7.6)	7 (15.2)	
Gestational age, wk				
<26	18 (16.1)	17 (25.8)	1 (2.2)	.002
26-31	8 (7.1)	4 (6.1)	4 (8.7)	
32-36	25 (22.3)	9 (13.6)	16 (34.8)	
≥37	58 (51.8)	33 (50.0)	25 (54.3)	
Unknown	3 (2.7)	3 (4.5)	0	
Maternal age, y				
<21	6 (5.4)	4 (6.1)	2 (4.3)	.13
21-25	24 (21.4)	13 (19.7)	11 (23.9)	
26-30	27 (24.1)	20 (30.3)	7 (15.2)	
31-35	25 (22.3)	17 (25.8)	8 (17.4)	
36-40	13 (11.6)	6 (9.1)	7 (15.2)	
≥40	6 (5.4)	1 (1.5)	5 (10.9)	
Unknown	11 (9.8)	5 (7.6)	6 (13.0)	
Course classification^c^				
Neonatal	64 (57.1)	42 (63.6)	22 (47.8)	.41
Known condition	37 (33.0)	18 (27.3)	19 (41.3)	
First presentation	9 (8.0)	5 (7.6)	4 (8.7)	
Unknown	2 (1.8)	1 (1.5)	1 (2.2)	
Site of death				
Inpatient	104 (92.9)	61 (92.4)	43 (93.5)	.84
NICU	86 (76.8)	52 (78.8)	34 (73.9)	
CVICU	8 (7.1)	5 (7.6)	3 (6.5)	
PICU	6 (5.4)	2 (3.0)	4 (8.7)	
Inpatient ward	1 (0.9)	0	1 (2.2)	
CCU	3 (2.7)	2 (3.0)	1 (2.2)	
ED	2 (1.8)	1 (1.5)	1 (2.2)	
Hospice	3 (2.7)	2 (3.0)	1 (2.2)	
Home	1 (0.9)	1 (1.5)	0	
Unknown	2 (1.8)	1 (1.5)	1 (2.2)	
Age at death, d				
0-4	24 (21.4)	16 (24.2)	8 (17.4)	.16
5-27	35 (31.3)	21 (31.8)	14 (30.4)	
28-89	30 (26.8)	16 (24.2)	14 (30.4)	
90-179	11 (9.8)	6 (9.1)	5 (10.9)	
180-364	10 (8.9)	5 (7.6)	5 (10.9)	
Unknown	2 (1.8)	2 (3.0)	0	
Cause of death				
Congenital malformations, deformations, chr anomalies	41 (36.6)	17 (25.8)	24 (52.2)	.002
Affected by maternal complications of pregnancy	2 (1.8)	2 (3.0)	0	
Disorders associated with short gestation and low birth weight	21 (18.8)	20 (30.3)	1 (2.2)	
Sudden infant death syndrome	5 (4.5)	3 (4.5)	2 (4.3)	
Accidents (unintentional injuries)	1 (0.9)	1 (1.5)	0	
Affected by complications of placenta, cord and membranes	4 (3.6)	4 (6.1)	0	
Neonatal hemorrhage	1 (0.9)	0	1 (2.2)	
Respiratory distress of newborn	1 (0.9)	1 (1.5)	0	
Bacterial sepsis of newborn	2 (1.8)	1 (1.5)	1 (2.2)	
Intrauterine hypoxia and birth asphyxia	3 (2.7)	3 (4.5)	0	
All other causes	31 (27.7)	14 (21.2)	17 (37.0)	
Pregnancy, labor, and delivery complications				
Preterm labor	51 (45.5)	30 (45.5)	21 (45.7)	.99
Cesarean delivery	67 (59.8)	38 (57.6)	29 (63.0)	.71
Oligohydramnios	4 (3.6)	2 (3.0)	2 (4.3)	.72
Polyhydramnios	13 (11.6)	3 (4.5)	10 (21.7)	.009
Placental abruption	6 (5.4)	6 (9.1)	0	.04
Maternal				
Infection	25 (22.3)	20 (30.3)	5 (10.9)	.03
Diabetes	10 (8.9)	5 (7.6)	5 (10.9)	.57
Hypertension	13 (11.6)	8 (12.1)	5 (10.9)	.85
Drug use	4 (3.6)	1 (1.5)	3 (6.5)	.17
Multiple gestation	11 (9.8)	8 (12.1)	3 (6.5)	.35

Abbreviations: CCU, critical care unit; chr, chromosome; CVICU, cardiovascular intensive care unit; ED, emergency department; NA, not applicable; NICU, neonatal intensive care unit; PICU, pediatric intensive care unit; WGS, whole-genome sequencing.

^a^ A genetic disease was identified in 46 infant deaths.

^b^ The race and ethnicity of infants were classified by parents. Race and ethnicity options were defined by the electronic health record. Infants with a racial and ethnic classification of “other” were those whose parents did not categorize them as Asian, American Indian or Alaska Native, Black or African American, Hispanic, Native Hawaiian or Pacific Islander, and non-Hispanic White and included multiracial infants.

^c^ Course classification: neonatal: death occurred at fewer than 27 days of life. Known condition: multiple hospitalizations or extended hospital stay with a known diagnosis. First presentation: first presentation or admission in a previously healthy child.

### Comparison of Genetic Diseases in Infant Deaths and Survivors

To further understand genetic determinants of infant deaths, we compared all 112 infants who died with all 434 surviving infants who underwent diagnostic WGS as inpatients at the same health system during the same period^26,27,28,29,30,31,32,33,36^ (Figure). There were no differences in sex, race, or ethnicity between infant deaths and survivors with or without underlying genetic diseases (eTables 1 and 2 in Supplement 1). However, single-locus genetic diseases were more common (46 of 112 [41%]) among infants who died than survivors (114 of 434 [26%]; *P* < .01; eTable 1 in Supplement 1). A literature review identified treatments that can improve outcomes for 54of the 114 (47%) genetic diseases in survivors, which was not significantly more than for the 47 genetic diseases in 46 infant deaths (19 [40%]; Table 1; eTable 3 in Supplement 1).^38,39,40,41^

The genetic diseases identified in infants who died and those who survived differed (Table 1; eTable 3 in Supplement 1). Only 6 of 148 (4%) identified disorders occurred in survivors and deaths, representing 20 of 178 infants (11%): *KMT2D*-Kabuki syndrome 1 (MIM#147920) occurred in 6 infants, of whom 5 survived; *CHD7*-CHARGE syndrome (coloboma of the eye, heart defects, atresia of the choanae, retardation of growth, genital abnormalities, and ear abnormalities; MIM#608892) occurred in 5 infants, of whom 3 survived; and chromosome (Chr) 22q11del-DiGeorge syndrome (MIM#188400) occurred in 3 infants, of whom 2 survived. These disorders are characterized by a spectrum of severity, and the association of phenotypic severity with risk of death is also likely significant. Thus, the specific genetic etiology had prognostic value (positive predictive value for death, 39 of 46 [85%]; positive predictive value for survival, 101 of 114 [89%]).

Genetic disease heterogeneity was greater among infant deaths than survivors. Thus, 45 of 47 diseases (95.7%) were unique to a single patient, vs 109 of 131 diseases (83.2%), respectively (*P* < .05; Table 1; eTable 3 in Supplement 1). Neither the mode of inheritance, variant type, nor predicted variant consequence differed between infant deaths and survivors (Table 1; eTable 3 in Supplement 1). Including structural variants, 32 of 47 disorders (68.1%) associated with infant deaths had autosomal dominant inheritance compared with 90 of 131 diseases (68.7%) in infant survivors. The high proportion of dominant disorders may reflect reduced reproductive fitness among infants who died and survived. Most variants affected single nucleotides (42 of 60 [70%] in infant deaths and 96 of 162 [59.3%] in infant survivors). The most common variant consequence was missense. It accounted for 30 of 60 variants (50%) in deaths and 67 of 162 variants (41.4%) in survivors. One of 46 infants who died and 12 of 114 infants (10.5%) who survived had 2 genetic diseases (*P* = .11).

### Death Certificate Review

Death certificates and EHRs were reviewed for 105 of the 112 deaths (93.8%) of infants who underwent WGS, including 45 of the 46 with genetic disease diagnoses. The remaining 7 death certificates could not be retrieved. Only 16 of 45 death certificates (36%) disclosed an underlying genetic etiology (Table 3). Eight of 14 genetic diseases identified postmortem had available treatments that could improve outcomes. Review of the EHRs revealed anticipatory clinical features in 5 of 8 deaths. Infant death might have been avoided had those features prompted rapid, diagnostic WGS at time of admission to the intensive care unit (ICU): *SCN1A*-developmental epileptic encephalopathy (DEE) type 6 (DEE6; MIM#619317) and *PCDH19*-DEE9 (MIM#300088) were identified in neonates 119 and 192 postmortem, respectively. These genetic epilepsies cause neonatal seizures that are often refractory to the standard antiseizure medications used in newborns.^38,39,40,41,42,43^ They had been admitted to the regional neonatal ICU with encephalopathy and seizures and died of respiratory failure on day of life (DOL) 9 and 6, respectively. They both received a clinical diagnosis of hypoxic ischemic encephalopathy. While both were delivered at term by cesarean delivery for decreased fetal movement, there was not an observed hypoxic event. Rapid WGS at neonatal ICU admission may have avoided misdiagnosis and potentially iatrogenic treatment for hypoxic ischemic encephalopathy while enabling treatment with specific antiepileptic drugs for DEE6 and DEE9.^38,39,40,41,42,43,44,45,46^

**Table 3.** Comparison of Death Certificates With WGS Findings in 45 Infant Deaths^a^

Patient No.	Affected locus	Immediate cause	Contributing 1	Contributing 2	Underlying cause	Other significant conditions associated with death	Operations performed
101	*KMT2D*	Cardiorespiratory failure	Herpes encephalitis	NA	NA	Kidney failure, liver failure, coagulopathy	NA
107	*PKHD1*	Cardiorespiratory arrest	Respiratory insufficiency	Pulmonary hypoplasia	Polycystic kidney disease	Severe hypoxic ischemic encephalopathy	NA
108	Chr1q dup	Cardiorespiratory failure	Partial trisomy 21	NA	NA	Abnormal chromosomes	NA
114	Trisomy 21	Respiratory failure	Hydrops fetalis	NA	NA	Atrioventricular canal, hypotension, Suspected trisomy 21	NA
119	*SCN1A*	Respiratory failure	Hypoxic ischemic enceph	Intraventricular hemorrhage	NA	Pneumothorax, cerebral edema, seizures, acidosis	NA
121	Chr17q11.2 del	Cardiogenic shock	Hypovolemic shock	Severe hypoxic ischemic enceph	Complex single ventricle, biventricular dysfunction	NA	ECMO, central venous placement, balloon septostomy, atrial septectomy
122	*NFKB1*	Citrobacter koseri meningitis	NA	NA	NA	NA	NA
124	*SUOX*	Cardiorespiratory failure	*E coli* bacteremia, diffuse brain injury	Refractory seizures	NA	Hypoxic ischemic enceph	NA
126	*NIPBL*	Respiratory failure	Cardiac failure	Cong heart dis	Cornelia De Lange syndrome	Microcephaly, failure to thrive	Nissen fundoplication, pulmonary artery banding
128	*CHD7*	Respiratory failure	Choanal stenosis	CHARGE syndrome	NA	Cleft palate, wound dehiscence, congenital stridor, tracheoesophageal fistula	Intubation, gastrostomy tube
133	*AUTS2*	Respiratory failure	Necrotizing cong *E coli* pneumonia	NA	NA	Pulmonary hypertension, kidney failure, seizures, chronic ventriculomegaly	Laparoscopic fundoplication with gastrostomy
138	*RYR1*	Respiratory failure	Dehydration	Poor feeding	Enceph	Aspiration, susp neurological condition	Laryngoscopy/bronchoscopy
141	*GATA6*	Respiratory failure	Cong diaphragmatic hernia	Complex cong heart dis	NA	NA	NA
142	*FGFR3*	Respiratory failure	Thanatophoric dwarfism	NA	NA	NA	NA
153	Chr 14q31q32 del	Respiratory failure	Hydrops fetalis	Chromosome 14q del	NA	Liver dysfunction, intraventricular hemorrhage	Peritoneal drain placement, skin biopsy
155	*CHD*7	Cardiorespiratory failure	Cranial nerve IX and X palsies	Mild Ebstein anomaly	CHARGE syndrome	NA	NA
158	Chr 17q12 del	Respiratory failure	Thanatophoric dwarfism	NA	NA	Persistent PH, hydrocephalus, abnormal electroencephalo	NA
160	*TAZ*	Heart failure	Dilated cardiomyopathy	Barth syndrome	TAZ variant	NA	NA
162	*SOX9*	Respiratory failure	Severe tracheobronchomalacia	Campomelic dysplasia	NA	Feeding problem, cardiopulmonary resuscitation	Tracheostomy, gastrostomy
166	*ARID1B*	Cardiorespiratory failure	Cong diaphragmatic hernia	NA	NA	PH, pneumonia, cong heart dis, intracardiac thrombus, Coffin-Siris syndrome	Repair cong diaphragmatic hernia and cong heart dis, ECMO
168	Chr 12q21q22 del	Respiratory failure	Pulmonary hypoplasia	Kidney failure due to polycystic kidney disease	Hypoxic ischemic enceph	Prune belly	Peritoneal dialysis catheter placement
170	*ANK2*	Anoxic brain injury complications	Resuscitated sudden infant death syndrome	NA	NA	NA	NA
171	*FBN1*	Acute respiratory failure	Low cardiac output syndrome	Severe coarctation of aorta	NA	twin birth	Pulmonary artery banding
173	*B3GALT6*	Arthrogryposis multiplex congenita	Perisylvian syndrome	NA	NA	NA	NA
177	*SDHA*	Cardiac failure	Cardiomyopathy	Left ventricular noncompaction	Mitochondrial complex II def	26 wk Premature, chronic lung dis, bacteremia, PH	Patent ductus arteriosus ligation
178	*TRNT1 GPD1L*	Immunodeficiency, developmental delay due to mitochondrial disease	NA	NA	NA	Sideroblastic anemia	Gastrostomy tube
181	*NDUFV1*	Heart failure	Lactic acidosis	Unknown metabolic disorder	NA	Small for gestational age	NA
182	*MBD5*	Respiratory failure	Congenital tracheal atresia	NA	NA	Obstructed bowel	Exploratory laparotomy
183	*CACNA1C*	Undetermined	NA	NA	NA	NA	NA
184	*TAB1*	Severe hydrocephalus ex vacuo	NA	NA	NA	Liver failure, respiratory failure	NA
188	*SAMD9*	Cardiac arrest	Respiratory arrest	Septic shock	Enterobacter bacteremia	Bilateral thalamic hemorrhages	NA
189	*COL6A3*	Respiratory failure	Hydrops fetalis, bilateral pleural effusion	NA	NA	NA	NA
191	*TAB1*	Brain herniation	Hemorrhagic meningoencephalitis	Human herpesvirus 6	Cerebral edema, meningoencephalitis	Status epilepticus, acute respiratory failure	NA
192	*PCDH19*	Cardiorespiratory failure	Pulmonary hemorrhage	Meconium aspiration syndrome	NA	Acute kidney injury, severe hypoxic ischemic enceph	ECMO
194	*COQ2*	Respiratory failure	Cong metabolic disease	NA	NA	T-cell lymphopenia	NA
198	Trisomy 22 mosaic	Hypoxia due to cong heart dis	Multiple organ failure	Pulmonary hypertension	Tricuspid atresia	NA	Right and left heart catheterization
200	*TTN*	Respiratory insufficiency	Apnea	Fetal akinesia deformation sequence	NA	NA	NA
201	*MOCS1*	Cardiac failure	Metabolic acidosis	NA	NA	Molybdenum cofactor deficiency with brain injury	NA
203	Chr 22q11 del	NA	NA	NA	NA	NA	NA
205	*IKBKG*	Respiratory failure	Intraventricular hemorrhage	Coagulopathy	Pseudomonas sepsis	Seizures	NA
207	Chr11p15q25 mosaic ROH	Cardiorespiratory failure	Kidney failure	Beckwith-Wiedemann syndrome	NA	Chronic lung disease, adrenal insufficiency	Ligation patent ductus arteriosus
209	*UBE3A*	Respiratory failure	Cerebral dysgenesis	NA	NA	Intractable seizures, microcephaly	NA
210	*SIX3*	Respiratory failure	Hydrocephalus	NA	NA	Holoprosencephaly, seizures, *SIX3* variant	NA
211	Chr18p11q23 dup	Cardiorespiratory failure	Trisomy 18	NA	NA	Kidney failure	NA
212	PPA2	Cardiopulmonary arrest	Possible aspiration	NA	NA	NA	NA

Abbreviations: CHARGE, coloboma, heart defects, atresia choanae, growth retardation, genital abnormalities, and ear abnormalities; cong, congenital; def, defect; del, deletion; dis, disease; dup, duplication; ECMO, extracorporeal membrane oxygenation; enceph, encephalopathy; NA, not applicable; PH, pulmonary hypertension; susp, suspected; WGS, whole-genome sequencing.

^a^ One certificate missing all fields was excluded.

*NFKB1* common variable immunodeficiency with autoimmunity 12 (CVID12; MIM#616576) was identified postmortem in neonate 122, who died of *Citrobacter koseri* meningitis on DOL 9. There has been an association between CVID12 and neonatal infection, including *C. koseri,* and it is frequently managed with immunoglobulin replacement and antibiotic prophylaxis.^47,48,49^ Rapid WGS at neonatal ICU admission may have been followed by CVID12 diagnosis and treatment with intravenous immunoglobulin and promptly administered broad-spectrum antibiotics.^47,48,49^

Infant 183 died of cardiac arrest on DOL 123. *CACNA1C* long QT syndrome 8 (LQT8; MIM#618447) was identified postmortem. Rapid WGS at neonatal ICU admission may have been followed by collection of a specific family history, electrocardiography, and, if abnormal, βadrenoreceptor blockade.^50^

Infant 212 died following a bradycardic arrest in the emergency department on DOL 42. *PPA2* infantile sudden cardiac failure (ISCF; MIM#617222) was identified postmortem.^51^ There is a frequent association between ISCF and neonatal sudden cardiac death, and it has been treated with cardioverter defibrillator implant and heart transplant.^52^ Rapid WGS during either of 2 prior pediatric ICU admissions may have been followed by diagnosis and consideration of these interventions.^51,52^

### Classification of Cause of Death

We classified the cause of death of the 112 infants according to the US Centers for Disease Control Wide-ranging Online Data for Epidemiologic Research guidelines^53^ and single-locus genetic disease as a separate, single category (Table 4). In the latter, genetic disease was the leading cause of death (46 deaths [41.1%]). The proportion of the 10 leading causes of death that were reclassified as genetic disease varied widely: 24 of 40 deaths (58%) with malformations were reclassified as genetic diseases. Malformations, which had been the leading cause of death, was relegated to third after exclusion of genetic disease. Congenital diaphragmatic hernia was the only common malformation in which genetic diseases were underrepresented (1 of 5). The second leading cause of death was prematurity (21 deaths [19%]), of which only 1 (5%) was reclassified as genetic disease. SIDS was the third leading cause of death (4 deaths [4%]), of which 1 (of infant 170) was reclassified as a genetic disease. Two other infants (183 and 212) died of isolated cardiopulmonary arrest that could be characterized as SIDS. Five of the 10 leading causes of death were unchanged, with inclusion of genetic disease as a category (maternal complications of pregnancy, prematurity, incidental events, complications of placenta, cord and membranes, intrauterine hypoxia/birth asphyxia, maternal pregnancy complications, and incidental events and hemorrhage). Thus, genetic diseases were not associated with infant deaths with known nongenetic risk factors. Eighteen of 30 deaths (57%) classified as “all other” were reclassified as genetic disease.

**Table 4.** Relative Proportions of Leading Causes of Death in 112 Infant Deaths in San Diego County Before and After WGS

Characteristic	Infant death causes, No. (%)	
	Before WGS	After WGS
Genetic disease^a^	0	46 (41)
Correct death certificate	NA	18 (16)
Incomplete death certificate		28 (25)
Congenital malformations, deformations, and chromosomal abnormalities	41 (36)	17 (15)
Disorders associated with short gestation and low birth weight, not elsewhere classified	21 (19)	20 (18)
Sudden infant death syndrome	5 (4)	3 (3)
Newborn affected by complications of placenta, cord and membranes	4 (3)	4 (3)
Intrauterine hypoxia and birth asphyxia	3 (3)	3 (3)
Newborn affected by maternal complications of pregnancy	2 (2)	2 (2)
Bacterial sepsis of newborn	2 (2)	1 (1)
Accidents (unintentional injuries)	1 (1)	1 (1)
Neonatal hemorrhage	1 (1)	0
Respiratory distress of newborn	1 (1)	1 (1)
All other causes	31 (28)	14 (12)
Total	112 (100)	112 (100)

Abbreviations: NA, not applicable; WGS, whole-genome sequencing.

^a^ Genetic diseases were classified as a single category.

### Generalizability of Findings

This study examined EHRs and administrative data of 125 of 390 infants (51%) born in San Diego County from 2015 to 2020 and WGS of 112 infant deaths (12%) (Figure). To assess the generalizability of the WGS findings, we compared 28 demographic, maternal, and infant characteristics of the 112 infant deaths for which WGS was available with all remaining 199 infant deaths with EHRs between 2015 and 2020 (Figure; eTable 4 in Supplement 1). The latter infants either were not enrolled to undergo diagnostic WGS in a clinical study due to ineligibility or parental refusal and did not have DNA samples in the Rady biorepository. The WGS and no WGS infant deaths differed in the incidence of diagnosed genetic diseases (41% vs 20%, *P* = .01), site of death, and age at death (Table 1; eTables 4 and 5 in Supplement 1). The proportion of genetic diseases associated with chromosomal anomalies was higher among infants who did not undergo WGS (24 of 41 [58.3%]) than those who received WGS (10 of 46 [21.7%]; *P* < .001; Table 1; eTable 5 in Supplement 1). Infants who underwent WGS experienced fewer deaths in the emergency department (2% vs 9%) and fewer deaths on DOL 0 to 4 (21% vs 29%). These differences were expected because WGS was not ordered from the emergency department, nor typically on the day of admission. The proportion of genetic diseases with available treatments that could improve outcomes did not differ significantly between the 2 groups (Table 1; eTable 5 in Supplement 1). Genetic diseases were identified in 87 of the total set of 311 infant deaths (28%) with Rady Children’s EHRs, which was similar to a previous study.^23^

To further assess generalizability, we compared 19 demographic, maternal, and infant characteristics in all 784 infant deaths in San Diego County from 2015 to 2019 with all 276 infant deaths with available health records (eTable 6 in Supplement 1). Infant deaths with available health records differed in gestational age (less prematurity), site of death (fewer deaths at home or in the emergency department), age at death (fewer deaths on DOL 0-4), and cause of death (more deaths associated with congenital malformations, prematurity, and SIDS), delivery by cesarean delivery (greater), and multiple gestation (less).

## Discussion

In this cohort study, genome sequencing identified single-locus genetic diseases in a high proportion (41%) of 112 infants with available health records who died between 2015 and 2020. Three lines of evidence supported the association of genetic diseases in these deaths: 83% of diseases identified had previously been associated with childhood mortality, almost all were consistent with the cause of death listed on the death certificate, and 70% had been diagnosed with those genetic diseases by rapid WGS for diagnosis of a suspected genetic disease during an ICU admission. Genetic diseases were also the leading cause of infant death (28%) among all 311 infant deaths with available health records between 2015 and 2020, of whom two-thirds did not undergo WGS. Previous studies have reported genetic diseases in 13% to 75% of infant deaths in various subpopulations with various genetic tests.^15,16,17,18,19,20,21,22,23,24,25,26^ However, most of these studies were small, limited to SIDS, and used exome sequencing, and all were retrospective. In the current study, all infant deaths were examined by WGS, and most were prospectively followed since ICU admission.

This cohort was reasonably representative of San Diego infant deaths during this period, with the exceptions of underrepresentation of deaths at home, the emergency department, and on DOL 0 to 4. For example, newborns who died on DOL 0 were underrepresented because blood sampling was not performed. Underrepresentation of DOL 0 deaths was anticipated to decrease the proportion of infant deaths associated with genetic diseases since many such deaths were associated with severe congenital anomalies. An ongoing follow-up study may circumvent these limitations by expanding WGS to all San Diego County infant mortality between 2014 and 2020 with archived blood spots.

Treatments with potential to be positively associated with outcomes were available for 27 of 87 genetic diseases (31%) that were associated with infant death. There is evidence that rapid WGS in infants in ICUs with diseases of unknown etiology is effective for genetic disease diagnosis, which has been followed by a practice guideline from the American College of Medical Genetics and Genomics and Medicaid coverage policies in 6 US states.^15,18,27,28,29,30,31,32,33,54^ Genetic diagnosis by rapid WGS at time of ICU admission and prompt administration of available treatments might have prevented the deaths of 5 of 14 infants with anticipatory clinical features in whom genetic diseases were identified postmortem (infants 183 and 212, with *CACNA1C *long QT syndrome and *PPA2*-ISCF, respectively died of cardiac arrest; neonate 122 with *NFKB1*-CVID12 died of sepsis; and neonates 119 and 192 died with *SCN1A*-DEE6 and *PCDH19*-DEE9, respectively). A limitation of this study was the uncertainty associated with reconstruction of the counterfactual clinical courses in these 5 infants. Nevertheless, these data suggested that infant mortality could potentially be reduced by broad use of rapid, diagnostic WGS in infants at the time of ICU admission with clinical features suggestive of genetic disorders. Rapid diagnostic WGS was used in 501 of 125 605 infants (0.4%) treated at this hospital system during this period.^26,27,28,29,30,31,32,33^ The obverse of this is that it is likely that there is even greater underrecognition of genetic etiologies of infant mortality in regions where rapid diagnostic WGS is not available. A future, alternative, and comprehensive approach to avoid underdiagnosis of the approximately 600 genetic diseases with current, effective treatments may be newborn screening (NBS) by automated WGS.^34,35^ Such WGS-based NBS is envisaged to be in addition to, and not a replacement for, traditional NBS or indication-based rapid diagnostic WGS.^34,35^

We identified several attributes of genetic diseases associated with infant death. No single genetic disease predominated; disease locus heterogeneity was at least as great in infants who died as in those who survived. Almost all of the genetic diseases identified in infant deaths were absent from infants with comparable illness who survived. Genetic diseases were 1.6-fold more common in infants who died than infants with comparable illness who survived. Among all infant deaths, term delivery and polyhydramnios were overrepresented in those associated with genetic diseases, while prematurity, placental abruption, and maternal infection were overrepresented in those without genetic diseases. These data suggest that further comparisons of the clinical features and molecular diagnoses of infants with genetic diseases who died and those who survived may yield integrative, prognostic models of infant survival and death.

### Limitations

As noted previously, the major limitations of this study were relatively small sample size, representation of a single US county, uncertainty whether genetic diseases were associated with infant mortality, and uncertainty associated with counterfactual clinical courses had diagnosis and treatment occurred before death. The study design led to underrepresentation of deaths at home, the emergency department, and on DOL 0 to 4.

## Conclusions

The results of this cohort study suggest implications for national vital statistics. An underlying genetic etiology was not recorded in approximately two-thirds of death certificates of infants with genetic diseases. This was higher than rates of death certificate inaccuracies in pregenomic studies.^11,12,13,14^ Whole-genome sequencing materially changed the etiology of 4 leading causes of infant mortality (congenital malformations/chromosomal abnormalities, SIDS, sepsis, and respiratory distress) and “all others,” which together comprised 71% of deaths. We recommend inclusion of the molecular etiology in the national vital statistics of these 4 leading causes of infant mortality. This is likely to reprioritize public health and research programs to combat infant mortality. While WGS is increasingly being adopted as a first-tier diagnostic test in ill inpatient infants, substantial challenges to implementation remain, and the optimal breadth of testing is unclear.^15,18,29,30,31,32,33,55,56,57^ Assuming a 13% mortality rate among infants receiving rapid WGS, it would be necessary to sequence approximately 1.9% of infants treated at this hospital system to identify all at risk of infant mortality. Broader use of rapid diagnostic WGS in a learning health care system of genome-informed neonatology may be associated with substantially reduced US infant mortality.^27^

## Notes

Supplement 1.
eMethods.
**eTable 1.** Comparison of sex, race, and ethnicity of 112 San Diego County (SD) infant deaths and 434 SD infant survivors who received WGS between 2015 and 2020 **eTable 2.** Comparison of sex, race, and ethnicity of 46 SD infant deaths with genetic diseases and 114 SD infant survivors with genetic diseases by WGS between 2015 and 2020 **eTable 3. **131 single locus (Mendelian) genetic diseases identified by WGS in 114 of 434 SD infants who survived **eTable 4.** Demographic and clinical characteristics of 112 SD infant deaths who received WGS and 199 SD infant deaths who did not receive WGS between 2015 and 2020 **eTable 5.** Demographic and clinical characteristics of all SD infant deaths and the subset who had a Rady Children’s Hospital (RCH) electronic health record (EHR) between 2015 and 2019

Supplement 2.
Data sharing statement
