# Supplementary material for: Reclassification of the Etiology of Infant Mortality With Whole-Genome Sequencing
Source: JAMA Netw Open. 2023 Feb 9;6(2):e2254069. doi: 10.1001/jamanetworkopen.2022.54069 (PMC9912130; doi:10.1001/jamanetworkopen.2022.54069)
Supplement: Supplement 1. — eMethods. eTable 1. Comparison of sex, race, and ethnicity of 112 San Diego County (SD) infant deaths and 434 SD infant survivors who received WGS between 2015 and 2020 eTable 2. Comparison of sex, race, and ethnicity of 46 SD infant deaths with genetic diseases and 114 SD infant survivors with genetic diseases by WGS between 2015 and 2020 eTable 3. 131 single locus (Mendelian) genetic diseases identified by WGS in 114 of 434 SD infants who survived eTable 4. Demographic and clinical characteristics of 112 SD infant deaths who received WGS and 199 SD infant deaths who did not receive WGS between 2015 and 2020 eTable 5. Demographic and clinical characteristics of all SD infant deaths and the subset who had a Rady Children’s Hospital (RCH) electronic health record (EHR) between 2015 and 2019 [file jamanetwopen-e2254069-s001.pdf]

Supplemental Online Content

Owen MJ, Wright MS, Batalov S, et al. Reclassification of the etiology of infant mortality with whole-genome sequencing. *JAMA Netw Open*. 2023;6(2):e2254069. doi:10.1001/jamanetworkopen.2022.54069

eMethods.
eTable 1. Comparison of sex, race, and ethnicity of 112 San Diego County (SD) infant deaths and 434 SD infant survivors who received WGS between 2015 and 2020
eTable 2. Comparison of sex, race, and ethnicity of 46 SD infant deaths with genetic diseases and 114 SD infant survivors with genetic diseases by WGS between 2015 and 2020
eTable 3. 131 single locus (Mendelian) genetic diseases identified by WGS in 114 of 434 SD infants who survived
eTable 4. Demographic and clinical characteristics of 112 SD infant deaths who received WGS and 199 SD infant deaths who did not receive WGS between 2015 and 2020
eTable 5. Demographic and clinical characteristics of all SD infant deaths and the subset who had a Rady Children's Hospital (RCH) electronic health record (EHR) between 2015 and 2019

This supplemental material has been provided by the authors to give readers additional information about their work.

© 2023 Owen MJ et al. *JAMA Network Open.*

eMethods

RCH is the only children's hospital in San Diego County (SD). RCH provides outpatient services throughout SD. The RCH EHR is used at all locations. The RCH EHR is also used at satellite neonatal intensive care units (NICU) at eight general hospitals with delivery services. There are children's hospitals separate from RCH in the neighboring Orange and Riverside Counties. Pediatric services and NICUs that do not use the RCH EHR are provided at several general hospitals in SD. The 629 infant deaths in SD from 2015-2020 who did not have an RCH EHR were either seen at another hospital or died in the community without being seen at a hospital. The diagnostic rate among infant deaths who had received standard genetic tests (21%, 41 of 199) was lower than among those receiving WGS (45%, 46 of 102). This is consistent with previous analyses of the rates of diagnosis of WGS and standard genetic tests[1].

1. Clark MM, Stark Z, Farnaes L, Tan TY, White SM, Dimmock D, Kingsmore SF. Meta-analysis of the diagnostic and clinical utility of genome and exome sequencing and chromosomal microarray in children with suspected genetic diseases. NPJ Genom. Med. 2018 3:16.

© 2023 Owen MJ et al. *JAMA Network Open.*

eTable 1: Comparison of gender, race, and ethnicity of 112 SD infant deaths and 434 SD infant survivors who received WGS between 2015 and 2020.

	Infant Deaths	Infant Survivors	p-value
Infants	112	434	
Genetic disease diagnosis	46 (41.1%)	114 (26.3%)	**0.003**
Sex			0.08
Male	58 (51.8%)	265 (61.1%)	
Female	54 (48.2%)	169 (38.9%)	
Unknown	0 (0.0%)	(0.0%)	
Race / Ethnicity			0.52
Hispanic	48 (42.9%)	185 (42.6%)	
White - Non-Hispanic	34 (30.4%)	120 (27.6%)	
Black or African American	8 (7.1%)	25 (5.8%)	
Asian	8 (7.1%)	21 (4.8%)	
Native Hawaiian or Pacific Islander	1 (0.9%)	19 (4.4%)	
American Indian or Alaska Native	1 (0.9%)	3 (0.7%)	
Other	12 (10.7%)	61 (14.1%)	

eTable 2: Comparison of sex, race, and ethnicity of 46 SD infant deaths with genetic diseases and 114 SD infant survivors with genetic diseases by WGS between 2015 and 2020.

	**Infant deaths with genetic disease**	**Infant survivors with genetic disease**	**p-value**
Infants	46	114	
Sex			0.48
Male	22 (47.8%)	63 (55.3%)	
Female	24 (52.2%)	51 (44.7%)	
Unknown	0 (0.0%)	(0.0%)	
Race / Ethnicity			0.71
Hispanic	18 (39.1%)	49 (43.0%)	
White - Non-Hispanic	15 (32.6%)	35 (30.7%)	
Black or African American	4 (8.7%)	5 (4.4%)	
Asian	1 (2.2%)	3 (2.6%)	
Native Hawaiian or Pacific Islander	0 (0.0%)	5 (4.4%)	
American Indian or Alaska Native	1 (2.2%)	1 (0.9%)	
Other	7 (15.2%)	16 (14.0%)	

© 2023 Owen MJ et al. *JAMA Network Open.*

eTable 3: 131 single locus genetic diseases identified by WGS in 114 of 434 SD infants who survived. Abbreviations: AD: autosomal dominant; AR: autosomal recessive; Chr: Chromosome; CHARGE: coloboma, heart defect, choanal atresia, retarded growth and development, genital hypoplasia, ear anomalies; DEE: developmental epileptic encephalopathy; Def: Deficiency; Dx: diagnosis; FP: first presentation; Hem: hemizygous; Het: heterozygous; Hom: homozygous; KC: known condition; LP: likely pathogenic; N: neonatal; NK: Not Known; P: pathogenic; Syn.: Syndrome; VUS: variant of uncertain significance.

ID	Sex	Age at WGS (days)	Inheri-tance	Condition	Affected Locus 1	Variant 1	Affected Locus 2	Variant 2	Locus 3	Variant 3
500	F	359	AD	Early infantile epileptic encephalopathy 7	*KCNQ2*	c.875T>C; p.L292P				
501	F	333	AR	Familial hyperinsulinemic hypoglycemia 1	*ABCC8*	c.2506C>T; p.R836*				
502	F	316	AD	Visceral myopathy	*ACTG2*	c.593G>A, p.G198D				
506	F	211	AR	Argininosuccinic aciduria	*ASL*	c.706C>T, p.R236W	*ASL*	c.706C>T, p.R236W		
515	F	40	AD	Kabuki Syn. 1	*KMT2D*	c.14753delC p.P4918Lfs				
518	M	255	AD	Kabuki Syn. 1	*KMT2D*	c.3228_3230delGAA;p.K1077del				
522	M	239	AD	Alveolar Capillary Dysplasia with misaligned pulmonary veins	*FOXF1*	c.188G>T, p.S63I				
523	M	200	AD	Mowat-Wilson Syn.	*ZEB2*	c.656delG, p.G219Afs				
530	M	170	XLR	Neonatal severe encephalopathy	*MECP2*	c.789dupC, p.G264Rfs				
531	M	277	AR	Def. of Factor XIII subunit A1	*F13A1*	c.1352_1353delAT p.H451Rfs	*F13A1*	c.1352_1353delAT p.H451Rfs		
532	M	14	AD	AD Mental retardation 35	*PPP2R5D*	c.592G>A p.E198K				
533	M	67	AD	Split Hand/Foot Malformation Type 3	10q24.31-q24.32 dup	chr10:102941001-103430600				
540	M	240	AD,AD	NOONAN Syn. 8; MYOCLONIC JUVENILE EPILEPSY	*RIT1*	c.295T>G p.F99V	*EFHC1*	c.124C>T p.R42C		
543	F	2	AD	TRISOMY 21	21p13q22.3 dup	chr21:1-48129895 dup				
546	F	111	AR	CONGENITAL DISORDER OF GCOSYLATION It	*PGM1*	c.1561C>T p.R521*	*PGM1*	c.1561C>T p.R521*		
548	F	234	AD,AD	Distal Arthrogryposis2B; Fam.ArrhythmogenicR.ventricular dysplasia8	*TPM2*	c.307C>A p.Q103K	*DSP*	c.925C>T p.Q309*		
553	F	96	AD,AD	AUTOSOMAL VISCERAL HETEROTAXY 5; Del. OF 4Q28.2-Q28.3	*NODAL*	c.591C>A p.Y197*	4q28.2q28.3 del	chr4:128895760-132764848 del		
555	F	15	AD	LACRIMOAURICULODENTODIGITAL Syn.	5p12 del (*FGF10*)	chr5:44247363-44418819 del				
556	F	26	AD	COSTELLO Syn.	*HRAS*	c.34G>A p.G12S				
559	F	21	AD	SCHUURS-HOEIJMAKERS Syn.	*PACS1*	c.607C>T p.R203W				
566	M	40	AR	SPINAL MUSCULAR ATROPHY 1	5q13.2 del (*SMN1*)	chr5:70247540-70247820 del	5q13.2 del (SMN1)	chr5:70247540-70247820 del		
570	M	20	AR	METHYLMALONYL-CoA MUTASE Def.	*MUT*	c.850G>T p.G284*	*MUT*	c.2114T>G p.I705R		
571	M	19	XLR	COMBINED IMMUNODef., XL	*IL2RG*	c.676C>T p.R226C				
581	M	8	AR	ATHABASKAN SEVERE COMBINED IMMUNODef.	*DCLRE1C*	c.406G>A p.D136N	10p13 del (*DCLRE1C*)	chr10:14983601-15065700 del		
583	M	181	XLR	BARTH Syn.	*TAZ*	c.800C>T p.T267M				
585	M	4	AD	CYANOSIS, TRANSIENT NEONATAL	*HBG2*	c.202G>A p.V68M				
589	M	49	XLD	G6PD Def.	*G6PD*	c.1450C>T p.R484C				
591	M	42	AR	BIOTIN-THIAMINE-RESPONSIVE BASAL GANGLIA Dis.	*SLC19A3*	c.597dup p.H200Sfs	*SLC19A3*	c.597dup p.H200Sfs		
592	F	47	AD,AD	LUSCAN-LUMISH Syn.; NKX2-5 RELATED DISORDER	*SETD2*	c.5122C>T p.R1708*	*NKX2-5*	c.23C>T p.T8M		
593	F	11	AR,AD	CBLC Methylmalonic aciduria & Homocystinuria; Del. of 3Q11.2	*MMACHC*	c.615C>G p.Y205*	*MMACHC*	c.271dup p.R91Kfs	3q11.2 q12.3 del	chr3: 95560647-102369178 del
595	F	11	AR	PROPIONIC ACIDEMIA	*PCCB*	c.665G>C p.G222A	*PCCB*	c.896C>T p.P299L		
596	F	9	AR	COENZYME Q10 Def., PRIMARY, 1	*COQ2*	c.590G>A p.R197H	*COQ2*	c.151A>G p.M51V		

© 2023 Owen MJ et al. *JAMA Network Open.*

597	F	12	XLD	CORNELIA DE LANGE Syn. 5	*HDAC8*	c.110G>A p.R37Q				
603	F	116	AD	PRRT2-RELATED DISORDERS	*PRRT2*	c.916_934del p.A306*				
607	F	4	AD	CHARGE Syn.	*CHD7*	c.5693T>G p.L1898*				
618	M	291	AD	VON WILLEBRAND Dis., TYPE 2	*VWF*	c.3797C>T p.P1266L				
624	M	150	AD	COL7A1-RELATED DISORDERS	*COL7A1*	c.6082G>A p.G2028R				
628	M	138	XLR	XL Immunodysregulation, Polyendrocrinopathy & Enteropathy	*FOXP3*	c.1010G>A p.R337Q				
630	M	13	AR	HYPER-IGE RECURRENT INFECTION Syn., AR	*DOCK8*	c.1648C>T p.R550*	*DOCK8*	c.*198G>A		
632	M	24	XLR,AD	Creatine Transporter Def.; Noonan Syn.-Like W. Loose Anagen Hair	*SLC6A8*	c.945_949del p.F315Lfs	*SHOC2*	c.4A>G p.S2G		
635	M	79	AD	Chr. 1P36 Del. Syn.	1p36.33p36.32 del	chr1:1478152-3342297				
640	M	8	AD	AURICULOCONDYLAR Syn. 1	*GNAI3*	c.119G>T p.G40V				
648	M	193	AR	POMT1-RELATED MUSCULAR DYSTROPHY-DYSTROGCANOPATHIES	*POMT1*	c.198_200del p.P67del	*POMT1*	c.1364_1365del p.T455Rfs		
649	M	14	AD	Factor V Leiden	*F5*	c.1601G>A p.R534Q				
650	M	18	AD, AD	Kabuki Syn. 1 ; MUSCULAR DYSTROPHY, DUCHENNE	*KMT2D*	c.16391_16392insAG p.L5465Gfs	Xp21.1 del (DMD)	chrX:32790510-32854924 del		
654	M	238	XLR	Glycogen Storage Dis. IX	*PHKA2*	c.1387C>T p.Q463*				
656	F	157	AD	Prader Willi Syn.	15q11.2-q12 del	chr15:23684685-26108259 del				
658	F	129	AD	CHARGE Syn.	*CHD7*	del				
662	F	76	AD	MOWAT-WILSON Syn.	*ZEB2*	c.1297C>T, p.Q433*				
663	F	67	AD	Autosomal visceral heterotaxy 5	*NODAL*	c.824G>A, p.R275H				
667	F	58	AD	Tuberous sclerosis-1	*TSC1*	c.1498C>T, p.R500*				
677	F	8	AD	Early infantile epileptic encephalopathy 7	*KCNQ2*	c.727C>G, p.L243V				
678	F	4	AD	Chr. 19q duplication Syn.	19q13.42q13.43 dup	chr19:54192301-59118400 dup				
683	F	4	XLD	Turner Syn.	chrX monosomy	chr X del				
686	F	6	AD	Prader Willi Syn.	15q11.2-q13.1 del	chr15:22833478-28566610 del				
691	F	16	AD	Stickler Syn. 1	*COL2A1*	c.2908_2909dupCC, p.P971Hfs				
704	F	9	AR	MAPLE SYRUP URINE Dis.	*BCKDHB*	c.410C>T p.A137V	*BCKDHB*	c.212T>G p.M71R; c.249C>A p.N83K		
707	F	55	AD	PERMANENT NEONATAL DIABETES MELLITUS	*INS*	c.26C>G p.P9R				
708	F	2	AD	Chr. 6q24-q25 Del. Syn.	6q24.2-q25.1 del	chr6:144951601-150260400 del				
712	F	16	AD	COSTELLO Syn.	*HRAS*	c.34G>A p.G12S				
721	F	15	AD	DIGEORGE Syn.	22q11.21 del	chr22:18893883-21562619 del				
725	F	16	AD	BRUGADA Syn. 1	*SCN5A*	c.4534C>T p.R1512W				
726	F	48	AD	Renal hypodysplasia/aplasia 3	*GREB1L*	c.3194C>T p.T1065I				
728	F	75	AD,AD	Susc. to Hirschsprung Dis. 1 and Early Onset Breast Cancer	*RET*	c.712G>T p.E238*	*BRIP1*	c.1234_1235delGA p.E412Sfs		
729	F	4	AD	Benign Neonatal Epilepsy 1	*KCNQ2*	c.1051C>G p.L351V				
731	F	8	AD	Kabuki Syn. 1	*KMT2D*	c.3968dupG p.R1324*				
735	M	142	AR	Dursun Syn.	*G6CP3*	c.207dupC p.I70Hfs	*G6PC3*	c.199_218+1del21		
737	M	161	AR	ALPHA-1-ANTITRYPSIN Def.	*SERPINA1*	c.1096G>A; p.E366K	*SERPINA1*	c.1096G>A; p.E366K		
740	M	109	AD	Autosomal visceral heterotaxy 5	*NODAL*	c.778G>A, p.G260R				
743	M	91	AD	Williams-Beuren Syn.	*ELN* (7q11.23 del)	chr7:72521701-74158700 del				
750	M	50	AD,AD	ATRIOVENTRICULAR SEPTAL DEFECT 3; Protein S Def.	*GJA1*	c.1085G>A, p.R362Q	*PROS1*	c.233C>T p.T78M		
753	M	43	AR	PYRIDOXINE-DEPENDENT EPILEPSY	*ALDH7A1*	c.328C>T, p.R110*	*ALDH7A1*	c.1279G>C, p.E427Q		
757	M	15	AD	DiGeorge Syn.	22q11.21 del	chr22:18893883-21568208 del				
770	M	10	AD	TRANSIENT NEONATAL DIABETES MELLITUS 2	*ABCC8*	c.4591A>C, p.T1531P				
781	M	4	AD	NOONAN Syn. 1	*PTPN11*	c.1510A>G, p.M504V				
785	M	9	AD	19p12q13.11 dup	19p12q13.11 dup	chr19:23158251-37100999 dup				
787	M	4	AD	SOTOS Syn. 1	*NSD1*	c.5431C>T p.R1811*				
788	M	42	AD	MOWAT-WILSON Syn.	*ZEB2*	c.1387delG p.V463Ffs				

© 2023 Owen MJ et al. *JAMA Network Open.*

794	M	4	AR	DUBIN-JOHNSON Syn.	*ABCC2*	c.3399_3400delTT p.Y1134Cfs	*ABCC2*	c.3851G>A p.W1284*		
799	M	101	AD,AD	Hypobetalipoproteinemia, Chr 1q21 del Syn.	*APOB*	c.2988_2994delCGGGGAC p.G997Pfs	1q21.1-q21.2 del	chr1:146631123-147416271 del		
811	M	3	AD	Emanuel Syn.	der(22)t(11;22)(q23;q11)	chr11:116691508-134257793, chr22:17038511-20307516				
817	M	4	AD	Congenital heart defect	*NOTCH1*	c.1810delA p.I604Sfs				
819	M	4	AD	LYMPHEDEMA, HEREDITARY, IA	*FLT4*	c.3121C>T p.R1041W				
843	M	88	AR	SPINAL MUSCULAR ATROPHY	*SMN1*	del	*SMN1*	del		
854	F	10	AD	Noonan Syn. 1	*PTPN11*	c.802G>T p.G268C				
856	F	5	AR	ELLIS-VAN CREVELD Syn.	4p16.2 del (*EVC2*)	chr4:5583651-5588641 del	4p16.2 del (*EVC2*)	chr4:5698802-5703481 del		
858	F	6	AD	FOCAL DERMAL HYPOPLASIA	Xp11.23 del	chrX:48239001-48611100 del				
863	F	8	AR	MAPLE SYRUP URINE Dis., TYPE IA	*BCKDHA*	c.1234G>A p.V412M	*BCKDHA*	c.1234G>A p.V412M		
867	F	7	AD,AD	SBBYSS Syn.; CHR. 16P11.2 DUP. SYN.	*KAT6B*	c.4162C>T p.Q1388*	16p11.2 dup	chr16:29453701-30300900 dup	*KAT6B*	c.4729C>T p.R1577C
872	F	5	AD	Early infantile epileptic encephalopathy 11	*SCN2A*	c.781G>A p.V261M				
874	F	10	AD	TURNER Syn.	Xp22.33q28 del	chrX:1-155270560 del				
878	F	43	AD,AD	SIFRIM-HITZ-WEISS Syn.; MYBPC3-RELATED CARDIOMYOPATHIES	*CHD4*	c.2648C>T p.S883F	*MYBPC3*	c.1790G>A p.R597Q		
880	F	8	AR	PROPIONIC ACIDEMIA	*PCCA*	c.1899+4_1899+7del	*PCCA*	c.1899+4_1899+7del		
882	M	14	XLD,AD	G6PD Def.; Dehydrated Her. Stomatocytosis W. Pseudohyperkalemia, Perinatal Edema	*G6PD*	c.292G>A p.V98M	G6PD	c.466A>G p.N156D	*PIEZO1*	c.3988G>T p.A1330S
883	M	84	AD	LAMB-SHAFFER Syn.	12p12.1 del (*SOX5*)	chr12:23707629-23726071 del				
885	M	4	AR	Cong. Sucrase-Isomaltase Def.	*SI*	c.4825C>T p.R1609*	*SI*	c.4825C>T p.R1609*		
888	M	51	AD	ACTN2-RELATED DISORDERS	*ACTN2*	c.355G>A p.A119T				
892	M	51	AD	CHARGE Syn.	*CHD7*	c.807del p.A270Pfs				
894	M	35	AR	AICARDI-GOUTIERES Syn. 1	*TREX1*	c.1033_1050del p.P345_A350del	*TREX1*	c.1033_1050del p.P345_A350del		
896	M	10	AD	MONOSOMY 1P36 Syn.; DUPLICATION OF 3P26.3	1p36.32p36.31 del	chr1:1-7083300 del	3p26.3 dup	chr3:1-2483200 dup		
903	M	59	AD	MYASTHENIC Syn., CONGENITAL, 18	*SNAP25*	c.589C>T p.Q197*				
906	M	20	AD,AD	Del. OF Chr. 20P13; Del. OF Chr. 20Q13.33	20p13 del	chr20:1-802900 del	20q13.33 del	chr20:61475301-63025600 del		
907	M	14	XLD	G6PD DEF.	*G6PD*	c.653C>T p.S218F				
910	M	6	AD,XLD	Complex cortical dysplasia w. other brain malformations 1; G6PD Def.	*TUBB3*	c.298_300del p.N100del	*G6PD*	c.1058T>C p.L353P	*G6PD*	c.466A>G p.N156D
914	M	9	AD	POIRIER-BIENVENU NEURODEVELOPMENTAL Syn.	*CSNK2B*	c.367+1G>A				
917	F	362	AR	Progressive early-onset Encephalopathy W. Brain Atrophy & Thin Corpus Callosum	*TBCD*	c.967C>T p.R323*	*TBCD*	c.1340C>T p.A447V		
918	F	60	AD	IMMUNODef., COMMON VARIABLE, 13	*IKZF1*	c.546C>A p.C182*				
922	M	10	AR	CENTRAL CORE Dis., AR	*RYR1*	c.131G>A, p.R44H	*RYR1*	c.10501dupG, p.D3501Gfs		
923	M	92	AR	AR distal spinal muscular atrophy 1	*IGHMBP2*	c.547+1G>A	*IGHMBP2*	c.1273C>T, p.R425C		
924	M	153	AR	Ataxia-telangiectasia	*ATM*	c.2250G>A, p.K750=	*ATM*	c.2250G>A, p.K750=		
926	M	52	AD	Kabuki Syn. 1	*KMT2D*	c.6010C>T p.Q2004*				
929	M	16	AD,AD	VENOUS THROMBOSIS	*F2*	c.1787G>A p.R596Q	*BRCA1*	c.2706_2707dup p.C903Yfs		
931	M	117	AD	BARAITSER-WINTER Syn. 1	*ACTB*	c.629G>A p.R210H				
932	F	129	AR	PYRUVATE KINASE Def.	*PKLR*	c.1529G>A p.R510Q	*PKLR*	c.307del p.R103Afs		

© 2023 Owen MJ et al. *JAMA Network Open.*

eTable 4: Demographic and clinical characteristics of 112 SD infant deaths who received WGS and 199 SD infant deaths who did not receive WGS between 2015 and 2020.

© 2023 Owen MJ et al. *JAMA Network Open.*

	Infant Deaths, Received WGS	Infant Deaths, No WGS	p-value
Infants	112	199	
Genetic disease diagnosis	46 (41.1%)	40 (20.1%)	**0.01**
Sex			0.41
Male	58 (51.8%)	114 (57.3%)	
Female	54 (48.2%)	85 (42.7%)	
Race / Ethnicity			0.83
Hispanic	48 (42.9%)	83 (41.7%)	
White - Non-Hispanic	34 (30.4%)	56 (28.1%)	
Black or African American	8 (7.1%)	19 (9.5%)	
Asian	8 (7.1%)	14 (7.0%)	
Native Hawaiian or Pacific Islander	1 (0.9%)	7 (3.5%)	
American Indian or Alaska Native	1 (0.9%)	1 (0.5%)	
Other	12 (10.7%)	19 (9.5%)	
Gestational Age			0.21
Less than 26 weeks	18 (16.1%)	29 (14.6%)	
26 - 31 weeks	8 (7.1%)	27 (13.6%)	
32 - 36 weeks	25 (22.3%)	38 (19.1%)	
37+ weeks	58 (51.8%)	84 (42.2%)	
Unknown	3 (2.7%)	21 (10.6%)	
Maternal Age			0.04
Under 21	6 (5.4%)	12 (6.0%)	
21-25	24 (21.4%)	24 (12.1%)	
26-30	27 (24.1%)	52 (26.1%)	
31-35	25 (22.3%)	36 (18.1%)	
36-40	13 (11.6%)	23 (11.6%)	
40+	6 (5.4%)	2 (1.0%)	
Unknown	11 (9.8%)	50 (25.1%)	
Disease Course Classification			0.04
Neonatal	64 (57.1%)	100 (50.3%)	
Known Condition	37 (33.0%)	59 (29.6%)	
First Presentation	9 (8.0%)	37 (18.6%)	
Unknown	2 (1.8%)	3 (1.5%)	
Site of Death			**2.4E-05**
Inpatient	104 (92.9%)	177 (88.9%)	
NICU	86 (76.8%)	103 (51.8%)	
CVICU	8 (7.1%)	37 (18.6%)	
PICU	6 (5.4%)	12 (6.0%)	
Inpatient Ward	1 (0.9%)	4 (2.0%)	
CCU	3 (2.7%)	21 (10.6%)	
ED	2 (1.8%)	18 (9.0%)	
Hospice	3 (2.7%)	0 (0.0%)	
Home or Unknown	3 (0.9%)	0 (0.0%)	
Unknown	2 (1.8%)	4 (2.0%)	
Age at Death			**0.02**
0-4 days	24 (21.4%)	57 (28.6%)	
5-27 days	35 (31.3%)	43 (21.6%)	
28-89 days	30 (26.8%)	37 (18.6%)	
90-179 days	11 (9.8%)	24 (12.1%)	
180-364 days	10 (8.9%)	38 (19.1%)	
Unknown	2 (1.8%)	0 (0.0%)	
Cause of Death			0.05
Congenital malformations, deformations, Chr. anomalies	41 (36.6%)	77 (38.7%)	
Affected by maternal complications of pregnancy	2 (1.8%)	12 (6.0%)	
Disorders related to short gestation + low birth weight	21 (18.8%)	33 (16.6%)	
Sudden infant death syndrome	5 (4.5%)	26 (13.1%)	
Accidents (unintentional injuries)	1 (0.9%)	6 (3.0%)	
Affected by complications of placenta, cord + membranes	4 (3.6%)	4 (2.0%)	
Neonatal hemorrhage	1 (0.9%)	2 (1.0%)	
Respiratory distress of newborn	1 (0.9%)	2 (1.0%)	
Bacterial sepsis of newborn	2 (1.8%)	6 (3.0%)	
Intrauterine hypoxia and birth asphyxia	3 (2.7%)	2 (1.0%)	
All other causes	31 (27.7%)	29 (14.6%)	
Pregnancy, Labor and Delivery Complications			
Preterm Labor	51 (45.5%)	94 (47.2%)	0.83
Cesarean section	67 (59.8%)	114 (57.3%)	0.78
Oligohydramnios	4 (3.6%)	5 (2.5%)	0.60
Polyhydramnios	13 (11.6%)	12 (6.0%)	0.10
Placental Abruption	6 (5.4%)	11 (5.5%)	0.95
Maternal infection	25 (22.3%)	27 (13.6%)	0.07
Maternal diabetes	10 (8.9%)	16 (8.0%)	0.79
Maternal hypertension	13 (11.6%)	17 (8.5%)	0.40
Maternal drug use	4 (3.6%)	9 (4.5%)	0.69
Multiple gestation	11 (9.8%)	13 (6.5%)	0.32

© 2023 Owen MJ et al. *JAMA Network Open.*

eTable 5: Demographic and clinical characteristics of all SD infant deaths and the subset who had an RCH EHR between 2015 and 2019.

	SD Infant Deaths	RCH EHR Infant Deaths	p-value
Infants	784	276	
Sex			0.85
Male	435 (55.5%)	155 (56.2%)	
Female	347 (44.3%)	121 (43.8%)	
Unknown	2 (0.3%)	0 (0.0%)	
Race / Ethnicity			0.26
Hispanic	369 (47.1%)	111 (40.2%)	
White - Non-Hispanic	200 (25.5%)	84 (30.4%)	
Black or African American	79 (10.1%)	24 (8.7%)	
Asian	49 (6.3%)	18 (6.5%)	
Native Hawaiian or Pacific Islander	1 (0.1%)	8 (2.9%)	
American Indian or Alaska Native	6 (0.8%)	2 (0.7%)	
Other	80 (10.2%)	29 (10.5%)	
Gestational Age			**4.7E-17**
Less than 26 weeks	295 (44.6%)	34 (15.1%)	
26 - 31 weeks	66 (10.0%)	25 (11.1%)	
32 - 36 weeks	78 (11.8%)	48 (21.3%)	
37+ weeks	196 (29.7%)	101 (44.9%)	
Unknown	26 (3.9%)	17 (7.6%)	
Maternal Age			0.05
Under 21	58 (8.8%)	15 (6.7%)	
21-25	127 (19.2%)	36 (16.0%)	
26-30	159 (24.1%)	57 (25.3%)	
31-35	182 (27.5%)	42 (18.7%)	
36-40	98 (14.8%)	28 (12.4%)	
40+	24 (3.6%)	5 (2.2%)	
Unknown	13 (2.0%)	42 (18.7%)	
Site of Death			**4.4E-04**
Inpatient	663 (84.6%)	251 (90.9%)	
ED	82 (10.5%)	18 (6.5%)	
Hospice	2 (0.3%)	2 (0.7%)	
Home	34 (4.3%)	1 (0.4%)	
Unknown	3 (0.4%)	4 (1.4%)	
Age at Death			**8.9E-27**
0-4 days	467 (59.6%)	72 (26.1%)	
5-27 days	121 (15.4%)	71 (25.7%)	
28-89 days	84 (10.7%)	60 (21.7%)	
90-179 days	55 (7.0%)	30 (10.9%)	
180-364 days	57 (7.3%)	42 (15.2%)	
Unknown	0 (0.0%)	1 (0.4%)	
Cause of Death			**4.6E-12**
Congenital malformations, deformations, Chr. anomalies	198 (25.3%)	101 (36.6%)	
Affected by maternal complications of pregnancy	123 (15.7%)	13 (4.7%)	
Disorders related to short gestation + low birth weight	75 (9.6%)	51 (18.5%)	
Sudden infant death syndrome	51 (6.5%)	27 (9.8%)	
Accidents (unintentional injuries)	28 (3.6%)	7 (2.5%)	
Affected by complications of placenta, cord + membranes	35 (4.5%)	8 (2.9%)	
Neonatal hemorrhage	26 (3.3%)	3 (1.1%)	
Respiratory distress of newborn	13 (1.7%)	3 (1.1%)	
Bacterial sepsis of newborn	18 (2.3%)	6 (2.2%)	
Intrauterine hypoxia and birth asphyxia	14 (1.8%)	5 (1.8%)	
All other causes	203 (25.9%)	52 (18.8%)	
Pregnancy, Labor and Delivery Complications			
Cesarean section	272 (34.7%)	158 (57.2%)	**3.5E-15**
Multiple gestation	110 (14.0%)	21 (7.6%)	**6.0E-03**

© 2023 Owen MJ et al. *JAMA Network Open.*
