# Supplementary material for: Reclassification of the Etiology of Infant Mortality With Whole-Genome Sequencing
Source: JAMA Netw Open. 2023 Feb 9;6(2):e2254069. doi: 10.1001/jamanetworkopen.2022.54069 (PMC9912130; doi:10.1001/jamanetworkopen.2022.54069)
Supplement: Supplement 2. — Data sharing statement [file jamanetwopen-e2254069-s002.pdf]

Data Sharing Statement

Owen. Reclassification of the Etiology of Infant Mortality With Whole-Genome Sequencing. *JAMA Netw Open*. Published February 09, 2023. doi:10.1001/jamanetworkopen.2022.54069

Data
Data available: Yes
Data types: Deidentified participant data
How to access data: Deidentified genome sequences, metadata, and clinical data of infants who died will be shared upon request to skingsmore@rchsd.org. Deidentified genome sequences, metadata, and clinical data of infants who survived will also be shared upon request, subject to authorization by the Data Governance Committee of the Rady Children's Institute for Genomic Medicine. Availability of deidentified data for living children is subject to parental election in individual consent forms.
When available: With publication

Supporting Documents
Document types: None

Additional Information
Who can access the data: Deidentified genome sequences, metadata, and clinical data of infants who died will be shared upon request to skingsmore@rchsd.org. Deidentified genome sequences, metadata, and clinical data of infants who survived will also be shared upon request, subject to authorization by the Data Governance Committee of the Rady Children's Institute for Genomic Medicine.
Types of analyses: For infant mortality research
Mechanisms of data availability: With investigator support and a signed data access agreement
Any additional restrictions: Availability of deidentified data for living children is subject to parental election in individual consent forms.
